# Correlation between protein expression profiling of inflammation and bone metabolism in rheumatoid arthritis patients

**DOI:** 10.4314/ahs.v23i3.73

**Published:** 2023-09

**Authors:** Jie Wang, Jian Liu

**Affiliations:** 1 Department of Rheumatology and Immunology, First Affiliated Hospital of Anhui University of Traditional Chinese Medicine, Hefei 230038, Anhui Province, China; 2 Institute of Rheumatology, Anhui College of traditional Chinese Medicine, Hefei 230038, Anhui Province, China

**Keywords:** Rheumatoid arthritis, bone destruction, self-perception of patient, protein expression profiling

## Abstract

**Objective:**

In patients with rheumatoid arthritis (RA), osteoarthrosis significantly reduces self-perception. However, the intrinsic relationship between bone metabolism and SPP and cell activity at the molecular level remains unclear. The purpose of this study was to understand the relationship between RA bone metabolic indicators and immune inflammation-related proteins.

**Methods:**

A total of 30 patients with RA and 30 healthy controls were recruited. Four bone metabolism measures and nine proteins expression measures were collected from RA patients and healthy controls. Spearman Correlation Analysis and Logistic-regression Analysis were adopted for associations between bone metabolism and proteins.

**Results:**

We screened and verified 3 key proteins, namely interleukin-11 (IL-11), interleukin-17 (IL-17) and programmed cell death-2 (PD-L2) related to immunity and inflammation through microarray analysis. Levels of IL-2, IL-5, IL-11, IL-17, CTLA4, TNF-β were higher in RA patients than in the control group (P<0.05), meanwhile, the levels of IL-8, PD-L2, TNF-β and B7-2 were low in RA patients (P>0.05).The results of Spearman Correlation Test suggested that sharp score was positively correlated with age, CCP was positively correlated with RF, SDS score was positively correlated with RF, IL-17 was positively correlated with CCP, BGP was positively correlated with BALP, RANKL was positively correlated with BALP, VAS score was negatively correlated with CRP, TCM score was negatively correlated with SF-36 score.

**Conclusion:**

BALP, BGP, OPG, RANKL were strongly associated with immune inflammation-related proteins and poor SPP in RA patients, which can be used to predict poor SPP in RA patients, although the underlying mechanisms need to be further explored.

## Introduction

RA is a chronic inflammatory autoimmune disease characterized by failure of spontaneous resolution of inflammation with lifetime perseverance, becoming one of the major causes of disability in millions of people. It is mainly characterized with progressive erosion of cartilage in response to formation of pannus leading to chronic polyarthritis and joint distortion^1^. In patients with recent-onset RA, the presence of one or more erosions is a well-established marker of poor prognosis that requires treatment intensification^2^. Histological examination of RA pannus shows a number of osteoclasts on the surface of the destructed bone. RA synovial tissues produce a variety of proinflammatory cytokines and growth factors that may increase osteoclast formation, activity, and/or survival^3^. The nuclear factor B receptor activator factor (RANKL/RANK/OPG) system plays an important role in regulating the dynamic balance between bone destruction and bone formation^4^.

Erosions and joint space narrowing, which were usually assessed using SHS in therapeutic trials, correlate with long-term functional impairments^5^-^6^. The self-perception of patient (SPP) involve the personal beliefs that patients have about their illness and may influence health behaviours considerably, which affect the initiation, progression, outcomes and return of RA^7^. Currently, the internationally recommended that SPP of RA is evaluated using the Disease Activity Score in 28 joints (DAS28), the Visual Analogue Scale (VAS), the Self-rating Depression Scale (SDS), the Self-rating Anxiety Scale (SAS) and the MOS item short from health survey (SF-36) and these had become the most accepted evaluation method by the majority of clinicians^8^. The previous studies of our research group showed that SPP was closely related to disease activity indexes such as ESR, RF, CRP, CCP in RA, which suggested that the higher disease activity of RA, the poorer SPP of patients^9^.

In this study, antibody arrays were performed to screen candidate biomarkers by comparing the expression variation of 50 cytokine in serum from 10 RA patients and 10 controls. Larger samples validation by ELISA in 40 patients. This study verified the potential relationship between key immune inflammatory proteins and RA bone destruction, and found a new mechanism of action, which could clarify a new direction for the study of RA bone destruction and provide a new idea for the next drug treatment target.

## Materials and methods

### Subjects

Ten (10) RA patients were selected from the Department of Rheumatology and immunology, the First Affiliated Hospital of Anhui University of Traditional Chinese Medicine between August 2019 and December 2019 (RA group), while 10 normal controls were identified as healthy by the physical examination center of our hospital (Control group). All RA patients fulfilled the 2010 ACR/EULAR (American College of Rheumatology/European League Against Rheumatism) criteria for the classification of RA^10^. Excluded the patients who combination with other rheumatic diseases, and with serious organic diseases, pregnant and lactating women. This study was approved by the ethics committee of our hospital, and all patients provided written informed consent. There was no significant difference in age and sex between groups.

### Collection of serum sample

The whole blood of patients and normal people were collected from 5ml, placed at room temperature for 30-45 min, centrifuged at 3000-5000 RPM/min for 10 min, and serum was collected and stored at -80^o^C until use.

### Screening of differential proteins

A total of 20 serum samples from 10 RA patients and 10 controls, were subjected to Raybiotech antibody array with 50 cytokines proteins for the differential proteins screening.

### Validation with large samples by ELISA

According to the above criteria, 40 RA patients were selected for ELISA verification. All serum samples and kit components were equilibrated to room temperature before the assay. The detection procedure was in accordance with the manufacturer's instructions.

### Measurements

All of the participants enrolled were asked to fill in the DAS28, VAS, SDS, SAS, SF-36 under the guidance of clinical doctors, SF-36 consists of 8 dimensions, OPG, BALP, BGP, RANKL. All clinical measurements were performed by the clinical laboratory staff of our hospital. The clinical laboratory data such as erythrocyte sedimentation rate (ESR), high-sensitivity C-reactive protein (CRP), rheumatoid factor (RF), anti-cyclic citrullinated peptide antibody (CCP), immunoglobulins A (IGA), immunoglobulin G (IGG), immunoglobulin M (IGM), complement 3 (C3), and complement 4 (C4) and clinical characteristics are determined. This study was approved by the ethics committee of our hospital, and all participating patients provided written informed consent.

### Bioinformatics analysis

Principal component analysis (PCA) and gene ontology (GO) analyses identified differentially expressed proteins and their functions, respectively. Kyoto encyclopedia of genes and genomes (KEGG) enrichment analysis then identified significantly enriched functions and pathways with p-values<0.05. Hierarchical clustering analysis grouped samples and proteins based on the differential protein expression patterns. PCA (R package “ggbiplot”), Hierarchical clustering analysis (R package “gplots”), GO analysis (R package “clusterProfiler”) and KEGG enrichment analysis (R package “clusterProfiler”) were performed using the open-source program R (version 3.5.1).

### Apriori algorithm

All the data were processed by R-Studio Version 3.5.1. we used the apriori algorithm to analyse the association rules of the herbs, differential proteins, laboratory indexes. The apriori algorithm is a frequent itemset algorithm for mining association rules. We used it to illustrate the specific rules of Bone metabolic indexes and Immunoin-flammatory indexes in RA treatment. The formulae were as follows:

support (X - Y) = σ, $\frac{\left(\text{X}\cup \text{Y}\right)}{N}$

confidence (X -Y) = σ ( ), $\frac{\text{X}\cup \text{Y}}{\text{σ}\left(\text{X}\right)}$

lift (X -Y) = confidence $\;\frac{\left(\text{X}\to \text{Y}\right)}{\text{σ}\left(\text{Y}\right)}$

where X -Y is an association rule, X (Consequent) and Y (Antecedent) represent the set of XFC and herb items, o(X) is the frequency of itemset X, X U Y is the union of itemset X and Y, o(X U Y) is the frequency with which itemset X and itemset Y appear together, support (X- Y) is the frequency with which X and Y appear together, and confidence (X - Y) is the probability that itemset Y appears in the presence of X. The lift is the ratio of the probability of itemset Y appearing in the presence of X to the frequency of Y. Support and confidence are often used to eliminate meaningless combinations; lift is the validity of the rules^11^.

### Statistical analysis

ROC curves were constructed to evaluate the diagnostic value of proteins for RA patients using the R package “pROC”. The differences in levels of proteins between RA patients and normal controls were analysed using the Wilcoxon Rank Sum test.

The clinical characteristics of the two groups were analysed using the unpaired Student's t-test. All statistical data were analysed using R software (version 3.5.1) and GraphPad Prism software (version 8.0). In all cases, p-values<0.05 were considered as statistically significant.

## Results

### Basic characteristics of RA patients and HC

30 RA patients (2 males, 28 females: mean age of 55.63±11.61 years, silk time mean age of 15.67±8.67 years) and 30 healthy controls (4 males, 26 females: mean age: 51.67±16.50 years) were used as the study group. No significant differences in age or gender were identified between the two groups (p > 0.05).

### Comparisons of sharp score, immune inflammation indicators, bone metabolism indicators and SPP between RA patients and HC

Compared with the control group, BGP, OPG, RANKL, BALP, IL-11 and IL-17 were significantly increased in the RA group (P < 0.05), and the difference was statistically significant, while PD-L2 was significantly decreased (P < 0.05).

### Differentially expressed serum proteins in RA patients

**Figure 1:**
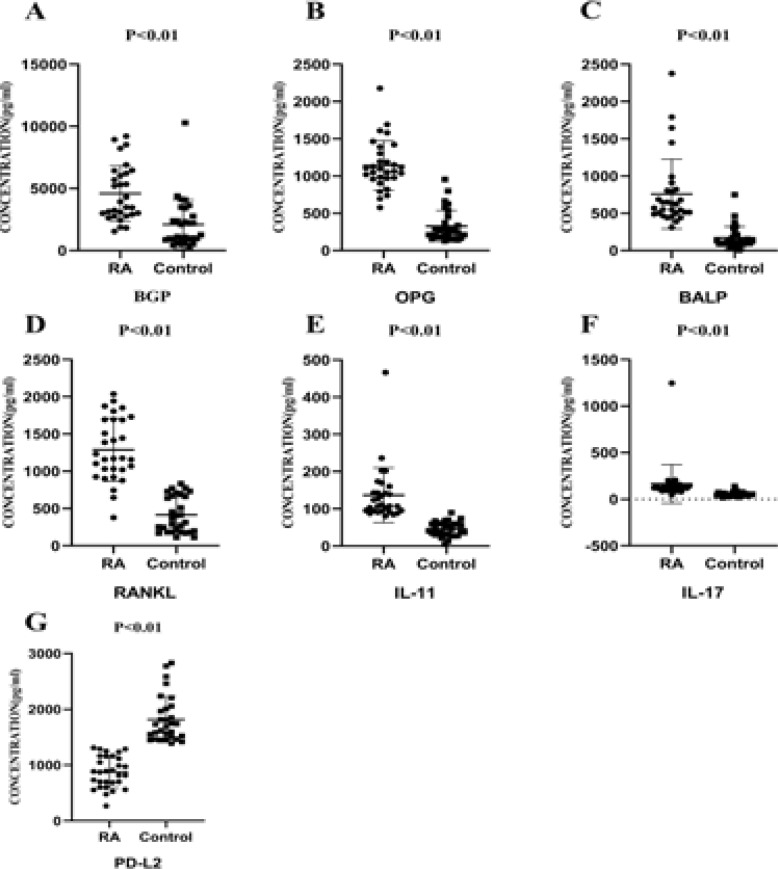
Comparisons of Sharp score, immune inflammation indicators, bone metabolism indicators and SPP Between RA Patients and HC

**Figure 2:**
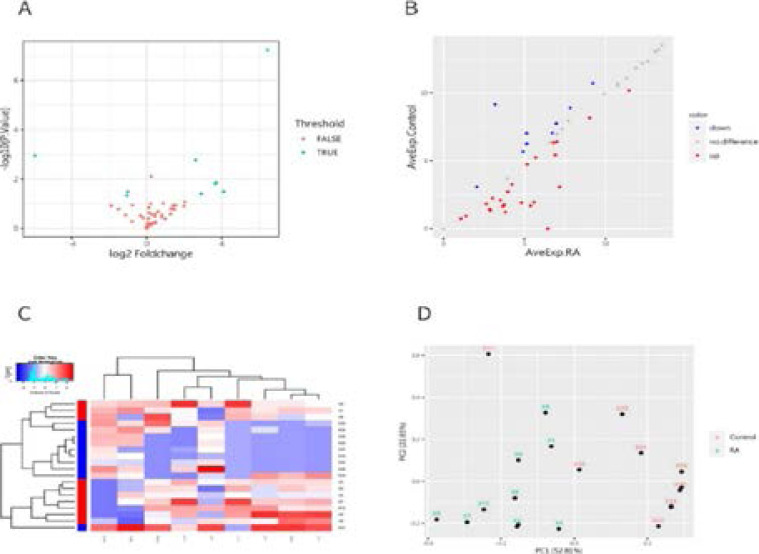
Protein expression profiles in RA and Control were detected by chip. (A) Volcano map of differentially expressed protein: the vertical line corresponds to two times above and below, and the horizontal line indicates that the p value is 0.05, while TRUSE indicates that the difference is significant. (B) Scatter plots of differentially expressed proteins: red: expression up-regulated; blue: expression down. (C) Hierarchical cluster analysis of significantly different protein expression in serum. Each row represents a sample and each column represents a protein. (D) PCA mapping of the 10 RA patients and 10 Control samples

The Raybiotech antibody array measured the concentration of 50 proteins in the serum from 10 RA patients and 10 controls. Twenty-four proteins were up-regulated, nine were down-regulated, and the rest were indistinguishable. A volcano plot depicting the proteins' expression levels shows that six proteins were significantly up-regulated in the RA patient group, including IL-2, IL-5, IL-11, IL-17, TNF-β and CTLA-4, while IL-8, PD-L2 , B7-2 was significantly down-regulated (logFC>log2(1.2), p-value<0.05) (Figures 1A and 1B, and Figure2).

A hierarchical clustering algorithm was used to cluster samples and differentially expressed proteins based on protein expression patterns (Figure 2C). The samples were divided into three main clusters. More specifically, the below cluster in Figure 1C contained seven patients, while the above cluster was comprised of both three patients and two controls. Although preliminary, these data suggest that the RA and controls groups can be distinguished from each other using antibody arrays and unsu-pervised clustering. The middle cluster was comprised of only controls. Principal component analysis (PCA) identified proteins that were differentially expressed across the two groups. A plot of the first two principal components (PC1 and PC2) for each sample shows a total variability of 52.81% and 22.65%, respectively (Figure 2D), thus implying that the protein expression patterns of SP patients and NOR are indeed different.

### Function of Differentially Expressed Proteins in RA patients

**Figure 3:**
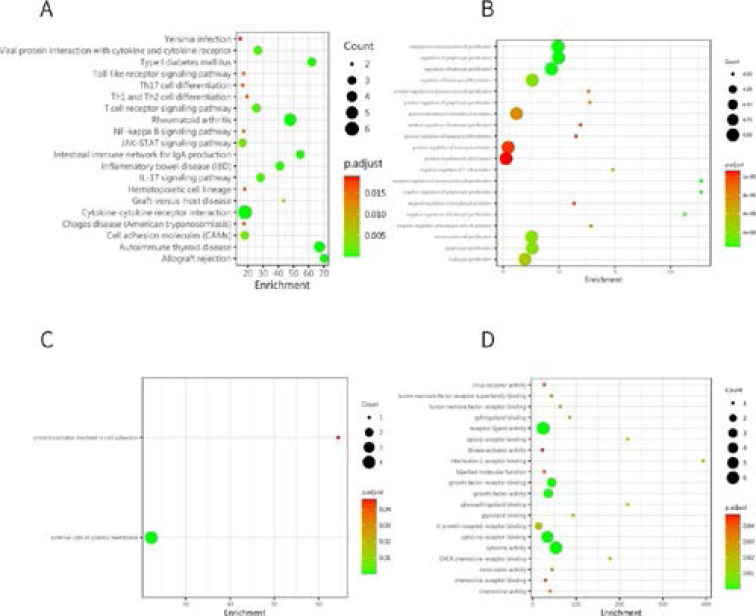
Bioinformatics analysis of different protein expression levels in RA patients and Control detected by microarray. (A-C) GO enrichment analysis of significantly different protein expression regarding (A) biological process, (B) molecular function, and (C) cellular component. (D) KEGG enrichment analysis of significantly enriched pathways in differentially expressed proteins

GO and KEGG enrichment analyses were performed to investigate the enriched functions and pathways of differentially expressed proteins associated with RA patients. GO enrichment analysis revealed that 292 significant functional terms were involved in the category of “biological process” (BP) function, 20 in the category of “molecular function” (MF) and 2 in the category of “cellular component” (CC) (Figures 3A, 3B and 3C). KEGG enrichment analysis revealed that differentially expressed proteins were significantly enriched in 22 pathways (Figure 3D). These enriched functional terms and signalling pathways may reflect rheumatoid arthritis disease pathogenesis.

### Verification of Selected differentially expressed proteins in RA patients

Levels of IL-2, IL-5, IL-11, IL-17, CTLA4, TNF-β were higher in RA patients than in the control group (P<0.05), meanwhile, the levels of IL-8, PD-L2, TNF-β and B7-2 were low in RA patients (P>0.05).

### Spearman correlation test between bone metabolism indicators, sharp score, immune inflammation indicators, SPP and differentially expressed proteins in RA patients

**Figure 5:**
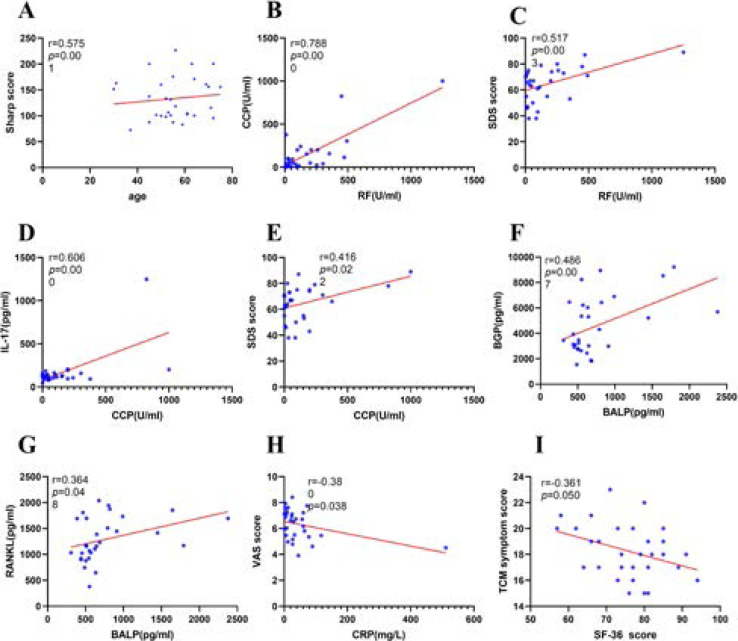
Spearman Correlation Test Between Bone Metabolism Indicators, Sharp Score, Immune Inflammation Indicators, SPP and Differentially Expressed Proteins in RA patients

The results of Spearman Correlation Test suggested that sharp score was positively correlated with age, CCP was positively correlated with RF, SDS score was positively correlated with RF, IL-17 was positively correlated with CCP, BGP was positively correlated with BALP, RANKL was positively correlated with BALP, VAS score was negatively correlated with CRP, TCM score was negatively correlated with SF-36 score (Figure 5A-I).

### ROC curve analysis of confirmed Proteins in RA patients

**Figure 4:**
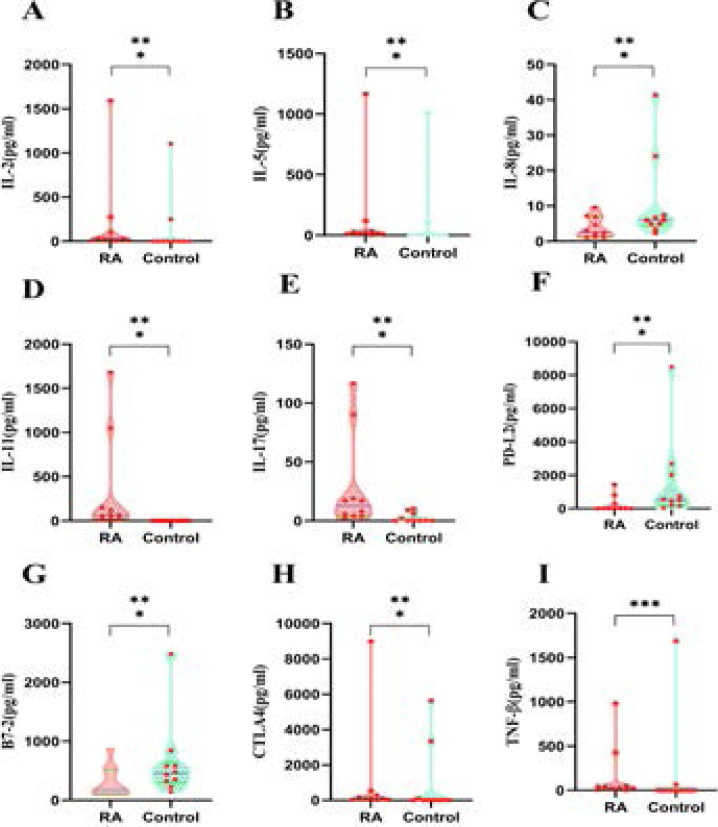
Verification of selected differentially expressed proteins in RA patients

ROC curves were constructed for the 9 differentially expressed proteins in serum to determine their sensitivity and specificity for diagnosing RA. ROC curve analyses revealed that, the proteins distinguished RA patients from Control with an area under the curve (AUC) > 0.70 (Figure 4). IL-11 and IL-17 and PD-L2 had the highest AUC at 1.000 and 0.830, respectively. Therefore, these proteins could be used as potential biomarkers for RA patients.

## Discussion

RA is a chronic progressive joint disease itself of autoimmune disease, common hands, knees, wrist, ankle joint shape change^12^, will accompany the inflammatory lesions at the same time, in addition the formation of the synovial inflammation and pannus is also the important pathological changes of the RA, including bone destruction is the most important factor in the RA patients with disabilities^13^. The symptoms of RA are mainly synovitis accompanied by destruction of articular cartilage and bone^13^, which is an important cause of the metabolic imbalance between osteoblast (OB) and osteoclast (OC), the increase of bone absorption and the destruction of bone tissue^14^. Normal human bone metabolism is a relatively balanced state, which is mainly maintained by bone destruction and bone resorption^15^. Bone destruction in RA patients interacts with and influences the course of disease, causing dysfunction of OC and OB, and imbalance of dynamic level, which further aggravates bone destruction^16^. The main regulatory system for this dynamic equilibrium is the receptor activator of nuclear factor-kappa B Ligand (RNAKL)/ receptor activator of nuclear factor-kappa B/osteoprotegerin (RANKL/RANK/OPG) system^17^. RANKL is currently the only cytokine found to promote the differentiation and maturation of osteoclast progenitor cells and inhibit cell apoptosis^18^.

We recruited 30 RA patients, in addition, 30 age- and sex-matched healthy participants were collected as healthy controls. There was no significant differences in age or gender were identified between the two groups (p > 0.05). The results of Spearman Correlation Test suggested that sharp score was positively correlated with age, CCP was positively correlated with RF, SDS score was positively correlated with RF, IL-17 was positively correlated with CCP, BGP was positively correlated with BALP, RANKL was positively correlated with BALP, VAS score was negatively correlated with CRP, TCM score was negatively correlated with SF-36 score All of the participants enrolled were asked to fill in the SPP scales under the guidance of clinical doctors. Statistical analysis of the SPP scales and clinical indexes obtained in RA patients showed significant differences between two groups. Compared with HC, SF36, MH of RA were significantly lower, while DAS28, VAS, SAS, SDS, BP were significantly higher than HC (P < 0.05).

The results of Spearman Correlation Test suggested that sharp score was positively correlated with age, CCP was positively correlated with RF, SDS score was positively correlated with RF, IL-17 was positively correlated with CCP, BGP was positively correlated with BALP, RANKL was positively correlated with BALP, VAS score was negatively correlated with CRP, TCM score was negatively correlated with SF-36 score. Similarly, in our study, we chose 10 RA patients and 10 healthy controls for antibody array. Finally, 50 significantly differentially expressed proteins were identified.

In contrast to HC, a total of 24 proteins were markedly upregulated and 9 proteins were significantly downregulated. We placed emphasis on GO analysis of proteins found that response to interleukin-17, regulation of in-terleukin-23 production, fibroblast activation was in BP, receptor regulator activity, receptor ligand activity was in MF. Moreover, top 2 pathways connected with functions of proteins in RA were defined by the KEGG analysis, rheumatoid arthritis, cytokine-cytokine receptor interaction were in proteins. We chose 9 differentially expressed proteins according to their expression distribution in each specimen by ELISA. As a consequence, the expression profile of IL-2, IL-5, IL-11, IL-17, TNF-β, CTLA-4 (P < 0.01) in the RA group were apparently higher than healthy control.

The expression profile of IL-8, PD-L2, B7-2 (p<0.01) in the RA group was apparently lower than in healthy control. Our results provide an insight into the potential pathogenesis of poor SPP in RA and provide a theoretical basis for the in-depth exploration of the function of poor SPP in RA. ROC curves of confirmed proteins showed that the levels of IL-2 (AUC=0.830), IL-5 (AUC=0.820), IL-11 (AUC=1.000), IL-17 (AUC=0.850), TNF-β (AUC=0.820), CTLA-4 (AUC=0.770), IL-8 (AUC=0.740), PD-L2 (AUC=0.830), B7-2 (AUC=0.740), which indicated that these proteins can be used as potential molecular markers for the diagnosis of poor SPP in RA. Finally, we performed Spearman Correlation test found that IL-11 was positively correlated with ESR, RF, negatively correlated with PF, MH, IL-17 was positively correlated with CCP, RF, DAS28, negatively correlated with PF, GH, SF, PD-L2 was negatively correlated with ESR, SAS, DAS28, positively correlated with SF. Additionally, results from Regression Analysis indicated that IL-11, IL-17 were risk factors for poor SPP in RA, while PD-L2 was protective factor for poor SPP in RA.

There are also many studies that just predicted the molecular biomarkers of RA by RNA-seq or microarrays. For example, Nys G et al applied a single targeted bottom-up proteomics LC-MS/MS to reveal serum amyloid A variants and alarmins S100A8-S100A9 as key plasma bio-markers of RA24. Sora M et al analysed serum samples of 18 RA patients and 18 healthy controls by LC-MS/MS to quantify serum proteins in RA patients. Then they selected 43 RA patients and 44 healthy controls to verified 6 significantly differentially expressed proteins.

Finally, they conclude that SAA4, gelsolin, and vitamin D-binding protein were validated as potential biomarkers of RA25. However, these two researches did not pay attention to SPP, they also did not associate the key proteins with SPP. Based on the results, we suggested IL-11, IL-17, PD-L2 as candidate biomarkers for effective predict poor SPP of RA patients. Nevertheless, there are some limitations in this study. First, we used 30 healthy controls and 30 RA patients for validation in ELISA. However, to validate the prediction of the 3 biomarkers set, a larger cohort study has to be performed. Second, comparison poor SPP between rheumatic disease and RA is needed to confirm whether these 3 biomarkers set has the ability to distinguish RA from other rheumatic diseases. We have plans to conduct the relevant experiments in future studies.

## Figures and Tables

**Table 1:** Basic Characteristics of RA Patients and HC

Items	RA(n=30)	Control (n=30)	P-Value
**Sex (woman/man)**	28/2	26/4	0.01
**Age**	55.63±11.607	51.67±16.495	0.286
**Course of the disease(year)**	15.67±8.672	NA	0.000
**Sharp score**	133.27±41.26	NA	0.000
**ESR (mm/h)**	50.05±27.60	NA	0.000
**hs-CRP (mg/L)**	49.29±92.09	NA	0.000
**RF(U/ml)**	177.01±250.60	NA	0.000
**CCP(U/ml)**	147.64±230.59	NA	0.000
**DAS28 score**	4.26±0.66	NA	0.000
**VAS score**	6.02±0.71	NA	0.000
**SAS score**	63.60±9.00	NA	0.000
**SDS score**	58.37±13.38	NA	0.000
**SF-36 score**	77.13±8.75	NA	0.000
**TCM symptom score**	17.90±1.97	NA	0.000
**The symptoms and signs**	18.43±2.93	NA	0.000

**Figure 6:**
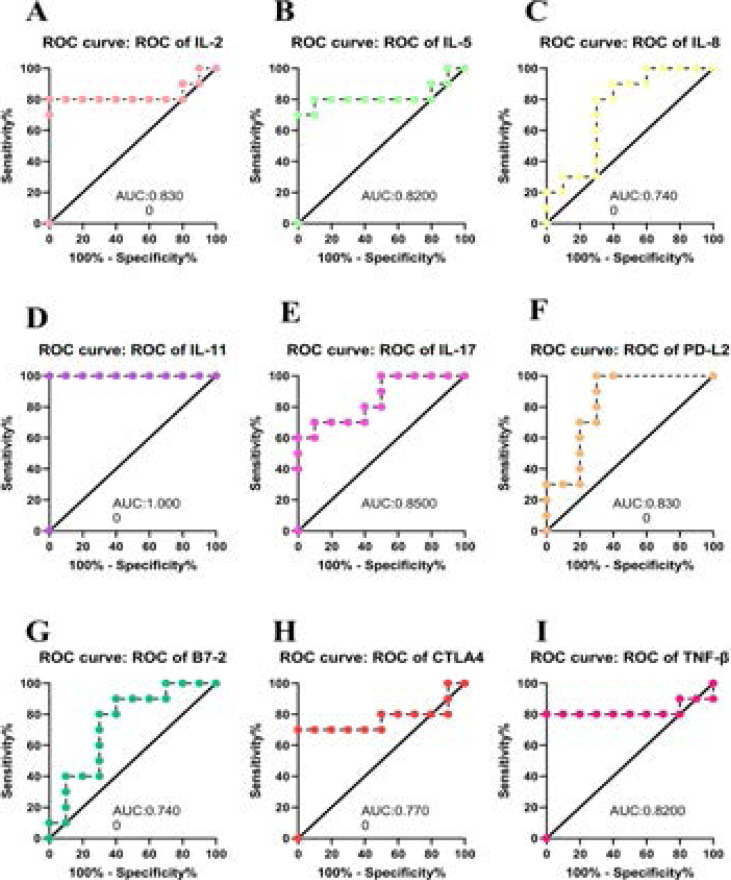
ROC curve analysis of differentially expressed proteins between RA patients and normal controls

## Data Availability

The data used to support the findings of this study are available from the corresponding author upon request.
